# Pregnancy vs. paycheck: a qualitative study of patient’s experience with employment during pregnancy at high risk for preterm birth

**DOI:** 10.1186/s12884-020-03246-7

**Published:** 2020-09-25

**Authors:** Sarahn M. Wheeler, Kelley E. C. Massengale, Konyin Adewumi, Thelma A. Fitzgerald, Carrie B. Dombeck, Teresa Swezey, Geeta K. Swamy, Amy Corneli

**Affiliations:** 1 Department of Obstetrics and Gynecology, Division of Maternal and Fetal Medicine, Duke University School of Medicine, 2608 Erwin Road #210, Durham, NC USA; 2 Diaper Bank of North Carolina, 1311 E. Club Blvd, Durham, NC USA; 3 Department of Population Health Sciences, Duke University School of Medicine, 215 Morris St. #210, Durham, NC 27701 USA; 4 Duke Clinical Research Institute, 200 Morris Street, Durham, NC 27701 USA

**Keywords:** Employment, Work, Preterm birth, High risk pregnancy

## Abstract

**Background:**

Pregnant women with a history of preterm birth are at risk for recurrence, often requiring frequent prenatal visits for close monitoring and/or preventive therapies. Employment demands can limit uptake and adherence to recommended monitoring and preterm birth prevention therapies.

**Method:**

We conducted a qualitative descriptive study using in-depth interviews (IDIs) of pregnant women with a history of preterm birth. IDIs were conducted by trained qualitative interviewers following a semi-structured interview guide focused on uncovering barriers and facilitators to initiation of prenatal care, including relevant employment experiences, and soliciting potential interventions to improve prompt prenatal care initiation. The IDIs were analyzed via applied thematic analysis.

**Results:**

We described the interview findings that address women’s employment experiences. The current analysis includes 27 women who are majority self-described as non-Hispanic Black (74%) and publically insured (70%). Participants were employed in a range of professions; food services, childcare and retail were the most common occupations. Participants described multiple ways that being pregnant impacted their earning potential, ranging from voluntary work-hour reduction, involuntary duty hour reductions by employers, truncated promotions, and termination of employment. Participants also shared varying experiences with workplace accommodations to their work environment and job duties based on their pregnancy. Some of these accommodations were initiated by a collaborative employee/employer discussion, others were initiated by the employer’s perception of safe working conditions in pregnancy, and some accommodations were based on medical recommendations. Participants described supportive and unsupportive employer reactions to requests for accommodations.

**Conclusions:**

Our findings provide novel insights into women’s experiences balancing a pregnancy at increased risk for preterm birth with employment obligations. While many women reported positive experiences, the most striking insights came from women who described negative situations that ranged from challenging to potentially unlawful. Many of the findings suggest profound misunderstandings likely exist at the patient, employer and clinical provider level about the laws surrounding employment in pregnancy, safe employment responsibilities during pregnancy, and the range of creative accommodations that often allow for continued workplace productivity even during high risk pregnancy.

## Background

In the US, preterm birth (PTB) and its complications are among the leading cause of neonatal death [1]. Women with a history of prior PTB are at increased risk for recurrence in future pregnancy. To reduce the risk of recurrent PTB, detailed care plans that require more frequent prenatal visits and monitoring are often recommended. For maximum benefit, preventive care must be initiated early and followed diligently throughout pregnancy. Recent data shows that one in seven US women receive inadequate prenatal care [2]. Despite these alarming rates and the urgent need for initiation and adherence to PTB prevention strategies, there are limited data on barriers to early prenatal care initiation in women with a history of PTB from the patient perspective. With the goal of uncovering patient perceived barriers to early initiation of prenatal care and developing interventions to mitigate barriers, we conducted a qualitative study of pregnant women at high risk for PTB.

In the US, over 50% of pregnant women work outside the home [3], and more than 20% of pregnant women are employed in low-wage jobs, earning less than $11.50 per hour [4]. Low-wage jobs often have limited flexibility in assigned work schedule and paid-time-off, making adherence to more frequent prenatal visits or monitoring even more challenging. In the US, there is also a well-documented race disparity in PTB such that non-Hispanic black women are 49% more likely than other racial/ethnic groups to experience PTB [5]. It is imperative to understand women’s employment experiences during high risk pregnancy, particularly experiences of non-Hispanic black women working in lower income wage jobs. These insights are critical to identify potential interventions that may alter the workplace experience to increase early initiation of care and adherence to PTB prevention. Here we describe findings from a larger qualitative study focused on barriers and facilitators to prenatal care in women with a history of prior PTB. As part of those interviews, women provided varied and important insights on the impact of employment on their high risk pregnancy care. The aim of this sub-study was to describe women’s experiences with employment during a pregnancy at high risk for PTB.

## Methods

### Parent study

The current manuscript is a sub-study of a larger parent study. The parent study was a qualitative descriptive study using in-depth interviews (IDIs). The IDIs were conducted between September 2018 and June 2019 in Durham, NC. The study was approved by the Duke Health Institutional Review Board (Pro00100090) on May 29, 2017. All participants provided written consent prior to participation. The parent study was focused on uncovering barriers and facilitators to initiation of prenatal care. The interview guide investigated numerous aspects of participants’ lives, ranging from how and when participants discovered they were pregnant, to how and when they disclosed their pregnancy to their partner and logistical barriers of scheduling appointments. The interview guide included questions that focused on the participant’s experience navigating pregnancy and employment (Supplemental file). The current analysis is focused on the data gathered from those interview questions.

### Eligibility criteria and recruitment procedures

Participants were recruited from the high risk obstetrical clinic of a large academic medical center. Participants were also recruited via a registry of women who delivered at the same institution after prior high risk pregnancy. This high risk obstetrical clinic is located in the southeastern US and serves approximately 2000 women per year, of which approximately 50% are non-Hispanic White and 40% are non-Hispanic Black. Women were recruited during their routine prenatal visit or via phone solicitation. Women were offered compensation for their time.

Women were eligible for study enrollment if they were currently or recently pregnant (within 2 months postpartum) and at high risk for PTB, defined as history of a prior spontaneous singleton PTB. We excluded women who did not speak English fluently. The current analysis also excludes women who were unemployed during their index pregnancy because the interview guide questions focused on employment were not applicable in that situation. Additionally, participation in the parent study was limited to women whose self-reported race and ethnicity is non-Hispanic Black or non-Hispanic White. We focused on these groups because the intent of the parent study was to descriptively compare experiences between the most prevalent racial/ethnic groups from the clinic population from which women were recruited.

### Data collection

Demographic information including age, race, insurance status and employment were obtained via electronic health record review and patient report. IDIs were conducted by trained qualitative interviewers in a private room near the clinic space or via phone at a time convenient to the participant. The interviewer followed a semi-structured interview guide focused on identifying barriers and facilitators to initiation of prenatal care and soliciting patient-perceived potential interventions to improve prompt prenatal care initiation. We asked participants to describe their current employment setting and experiences communicating with their supervisor about their pregnancy, scheduling shifts, and any pregnancy-related workplace accommodations. Interviews were audio-recorded with participant permission and transcribed verbatim.

### Analysis

Demographic data were analyzed and presented using descriptive statistics. The IDIs were analyzed via applied thematic analysis [6], by two analysts trained in qualitative data analysis. The codebook was developed incorporating structural and emergent codes. Structural codes were applied to the transcripts to organize participant responses based on the interview guide question to which the participant was responding and/or a related topic(s) spontaneously mentioned during the interview. The codebook was then expanded to include additional emergent codes associated within each structural code after reviewing the transcript passages associated with each structural code. We then applied the emergent codes to the transcripts. Inter-rater reliability procedures were conducted on 27% (*n* = 33) of interview transcripts with coding differences resolved by discussing individual instances until consensus was reached. Focusing on employment experiences, we then examined the narratives within the emergent codes to discover common themes among participant responses. Patterns across responses were identified within individual codes, then patterns across codes were identified to reveal salient themes. We first identified experiences that participants perceived asimpacting their abilities to maintain employment during a high risk pregnancy. We then organized those experiences into two categories based on whether the experiences influenced participants’ income or were related to the implementation of pregnancy-related workplace accommodations.

## Results

The current analysis includes 27 of the 33 women interviewed for the parent study. We excluded six women from the current analysis because they were not employed during their index pregnancy, and therefore they were not asked the employment questions during the interview. Participants (*N* = 27) were on average 29 years old, majority non-Hispanic Black (NHB) (74%), and most often publically insured (70%) (Table 1). Participants were involved in diverse fields of employment. Food services, childcare and retail were the most common occupations (Table 2). Our analysis identified two broad over-arching themes: negative implications of pregnancy on income and challenges related to workplace accommodations.

**Table 1:** Participant Characteristics (*n* = 27)

Participant Characteristics	
Age (mean, SD)	29.1 (5.1)
Self-Described Race (n,%)	Non-Hispanic Black (20, 74%)
	Non-Hispanic White (6, 22%)
	Mixed race (1, 4%)
Insurance status (n,%)	Private (7, 26%)
	Public (19, 70%)
	Uninsured (1, 4%)

**Table 2:** Study Participant Occupations

Employment	Study Population	Most Common Occupations among US Women by Rank [7]		
		All Pregnant Women	Pregnant Black Women	All Women
Food Service	8 (29%)	7		
Childcare (Daycare / Preschool)	5 (19%)			
Retail	5 (19%)	3	1	
Healthcare/Nurse	3 (11%)	5	2	1
Customer Service	2 (7%)	6	3	5
Real estate/property management	2 (7%)			
Biology Lab	1 (4%)			
Hotel Housekeeping	1 (4%)			
Other top ranked occupations not represented in the study cohort		1 Elementary teacher 2 Registered nurse 4 Administrative assistant	4 Personal care aides 5 Registered nurse	2 Administrative assistant 3 Elementary / Middle teacher 4 Managers, all other

### Income implications

Participants described multiple ways that they felt their income decreased due to their pregnancy. Some of the pay decreases were the result of reduced duty hours, while other participants described limitations due to unpaid leave (Table 3). Women working in non-hourly employment also said that pregnancy negatively impacted their income.

**Table 3:** Participant employment experiences

**Income Implications**	
Reduced duty hours	“They understood I worked another job besides them, so they didn’t want me to overwork myself so they would like have me come like, all the way to [street name] like, two days a week, whatever, which really wasn’t worth my time you know, just to go to the bank or whatever for the what, twenty dollars, ten, twenty dollars. That’s all I really made a week you know, for those two days [food service worker, age 31].”
	“That’s why my hours are cut now because I kept having to get off early, like take off on these days just to go to get my [prenatal care]. So that’s really the conflict [retail worker, age 25].”
Unpaid leave	“[Taking time off is] pretty much what was interfering and like I told them, I am pregnant, I do gotta receive prenatal care, so if you don’t feel I’m reliable because of that then, go ahead, do what you gotta do…. ‘cause my child’s life is more important than this freaking job. The same way I got this job, I can always find me another one… she tells me, I’m pregnant and I’m not reliable. Okay, that ain’t a good reason to let somebody go. That’s just discrimination on your part… [food service worker, age 31].”
	“Oh, it just you know, makes the paycheck a little smaller, but other than that, that’s it [retail worker, age 37].”
Consequences for non-hourly employees	“I usually take off, when I have to go in the morning I’ll do like from 9:00 to 11:00, so I try to do like two, three, about two hours. …the only thing it really messed with is like my adherence at work. My adherence and my, basically my adherence and attendance. It kind of messes with those… Adherence is just your time on the phone, time away from the phone, and being on the phone when you’re actually supposed to be on the phone [customer service, age 29].”
	“I would say, just because I am being monitored more closely [by my doctor], I do have a fair number of more doctor’s appointments than I did last time. I will say it has been a little tricky with the balance of you know, work and then having my workday kind of interrupted with an appointment. I never would skip my appointment just because I feel like I have to be at work, but there are some times where I feel you know, a little guilty, oh I’m missing that meeting or something like that. But like I mentioned before, my boss has never once said like, no you really need to be here, you can’t go to your doctor’s appointment you know, kind of thing. It’s more of a, I just personally feel a little guilty, yeah [scientist, age 32].”
**Workplace Accommodations**	
Collaborative employer/employee accommodations	“So, it’s usually, it’s a fairly loose working environment. I mean the schedules are pretty flexible and our boss is extremely understanding of just you know, family and personal needs and so we’ve kind of you know, as long as we’re there for you know, our eight hours a day and we’re actually making progress on our work, we kind of the ability to be pretty flexible with what we do [scientist, age 32].”
Employer initiated accommodations	“… we talked about when I get further along, closer to my due date, instead of me serving, we’ll move to a different position, it’s hosting, so it would still require me to be on my feet, but I wouldn’t be walking as much and it’s not a lot of lifting like, of trays and food and drinks and things like that. I would just be picking up things and wiping off \s and that stuff [food service worker, age 29].”
Medically recommended accommodations	“And I didn’t really work there too much longer after that ‘cause I was like you know, they don’t want to be supportive then, you know, they can just kick rocks and I can be happy with my healthy baby [food service worker, age 20].”

#### Reduced duty hours: participant initiated

Many of the study participants (*n* = 13) described income reduction due to decreased hours worked that they attributed to their pregnancies. Over half (*n* = 7) of the work hour reductions that were described resulted from participant-initiated requests for a reduced workload. Some (*n* = 4) participants requested to work shorter shifts and some (*n* = 3) chose to leave their jobs. Most often the participant-initiated duty hour reductions or employment terminations were linked to pregnancy discomfort. One of these participants, a food service worker, described reducing her hours at a job that did not allow for much sitting: “*I used to work full time, but now I work part time because it’s more of, I work in hospitality so I be on my feet a lot… now that I’m further along I experienced more lower back pain, under my stomach.”* Another participant, also working in food service said, *“I stopped working [at fast food restaurant] … when like, my back started hurting or I was being like, I was moving slower like, I wasn’t with production time and stuff like that.”* The participant also explained that the smells from certain foods cooking at the restaurant made her vomit during her shifts.

#### Reduced duty hours: supervisor initiated

Multiple participants (*n* = 6) described supervisors who reduced the number of hours participants worked despite their desire to continue working. For example, a participant working in retail described that her employer responded to her pregnancy by: “*Cutting my hours, that’s how she really responded to the whole [pregnancy] thing. She just cut my hours.”* Another participant, a food service worker, described a similar experience:

*“They didn’t know I was pregnant right away… they was like, ‘you know, you’re showing a little.’ So that’s when that came out. So that’s definitely, [fast food restaurant] changed my schedules, [from 7am to 4pm to] like 8 to 4, whatever you know, 10 to 4. They tried to sneak an 11 to 4.”*

There were also participants who expressed positive work experiences prior to their pregnancy, yet during their pregnancy they terminated their employment due to decreased earnings and truncated advancement. A participant working in food service commented, *“[Supervisor] was really like communicative and nice, but at the same time she was cutting my hours... so she was really nice about it, but at the same time, she knew that I needed the hours and she was still cutting them. So, that’s why I had to leave because I was not making any money.”* Another participant also a food service worker, who was excited for an upcoming promotion, described leaving her job due to unsupportive attitudes towards her pregnancy: *“I worked so hard you know, I fought for that spot… ‘cause I love working there, eating there, but [fast food restaurant] weren’t really supportive of pregnant women working there at all you know.”*

#### Unpaid leave

Less than one-third of the participants in our study were eligible for paid or partially-paid leave from their jobs. One participant, working in real estate, described a finite amount of time she could take off from work to attend prenatal visits before delivery and during postpartum recovery. She had to weigh when to use paid time-off:

*“I try not to use my PTO [paid time off] because you know you have to use PTO when you go on FMLA [Family and Medical Leave Act], before your short-term disability starts, so sometimes I just have to take the hit and not hit my forty hours, which means a lower check.*”

Some participants (*n* = 3) described situations in which unpaid leave was couched as a benefit to employees who were told they could return to work after delivery without having to reapply for their jobs. A food service worker explained she was told: “*Just a leave, basically like you’re just quitting and then you just get rehired*.” Another food service worker said she knew to expect being removed from the work schedule towards the end of her pregnancy: *“…so as far as my due date, I told him that I’m due in September and he knows like, when it gets close to August, he’ll actually be preparing to take me off the schedule until I’m ready to return to work*.”

#### Reduced income in non-hourly employees

Participants whose time at work was not scheduled in hourly shifts also experienced negative impacts to their take-home pay due to pregnancy. One participant, who worked for a small retail business, reported that her supervisor allowed her to make-up the missed work due to prenatal visits at another time so that her take-home pay was not impacted, but only during the business’ “*busy season*.” Otherwise, she had to take unpaid leave from work to attend prenatal visits. Another participant who works in real estate, had a flexible work schedule. However, since she was paid on commission, time away from work could still impact her take-home pay as she explained, “*If I’m not there then I’m just not there [to earn money].*”

Many of the participants who worked in childcare reported positive experiences because they were able to balance work with prenatal and postpartum care. For one participant, the timing of her pregnancy meant that she could avoid reduced work hours. As the childcare worker explained, she did not have to take unpaid leave to attend prenatal visits since she disclosed her pregnancy during the summer, prior to the finalization of teaching assignments for the current school year*. “I wanted to tell [supervisor] when my schedule was being like, figured out because I wanted to try and build in certain pockets of time when work was easier for me to get away for appointments.”* The participant further explained that she was then able to attend healthcare visits during her planning period and make-up the missed work time on her own to avoid any impacts to her take-home pay, since she requested the schedule changes before the school year started.

### Workplace accommodations

Participants discussed accommodations that their employers and coworkers made in their workplace environment or within job duties during their pregnancies. These accommodations were sometimes a collaborative effort between the participant and her employer; in other situations they were employer-initiated based on the employer’s perception of safe working conditions during pregnancy, and in other instances accommodations were medically recommended by an obstetrical care provider (Table 3).

#### Collaborative employer/employee accommodations

Some of the most positive employment experiences recounted were collaborative efforts between the participant and her employer to adjust the workplace environment and job duties allowing the participant to continue performing her job duties while feeling safe and supported. A healthcare worker described: “*They were very supportive. They told me that, you know, I wanted to do night shift and I wanted to do like an easier hall, like maybe rehab or something like that, and they were completely open to it; they were fine*.”

Another healthcare worker, whose job required several hours of sitting and typing, described how her employer helped to make her workstation more comfortable:

“… if I need to be off or whatever, if I needed any accommodations to my workspace like, I had to buy, I didn’t have to buy it, they reimbursed me for it, but just different, like back supports and footstools and stuff that I started needing later ‘cause I was sitting a lot you know, they reimburse for, but anything I mean anything I needed they were onboard for.”

#### Employer-initiated accommodations

In other situations, workplace restrictions stemmed from the employer’s perception of safe working conditions during pregnancy. For example, a real estate worker explained that her manager “*barely lets me carry the water*” even though her medical provider had not recommended any lifting restrictions. Another participant working in food service without any provider-documented restrictions, described how her employer frequently considered her pregnancy: “*They were strict with me of doing a lot of things. I couldn’t lift nothing, I couldn’t do too much bending like, kind of you know, they took my health into consideration, most definitely.*”

#### Medically recommended accommodations

Participants also described situations where their medical provider recommended modifications to their job duties. These medically recommended restrictions were met with varied reactions at the workplace. A participant working in retail described a very supportive response from her employer. She said, *“They’re supportive of me and they’ve been working with my accommodations… right now I’m on a twenty-five-pound weight restriction, so anything that would feel too heavy for me to lift, or to move, they actually do it for me.”* Other participants described more negative reactions from their employers upon requesting medically-recommended accommodations during the workday. Two participants recounted experiences in which they perceived that their employment was terminated due to a request for accommodations recommended by a medical provider. One participant shared her experience of being laid off from her job at a grocery store when she shared that her physician had recommended an accommodation to her job duties:

“*I was just laid off yesterday… due to my limits and restrictions from the doctors…. I was put on a ten-pound lifting limit, which is very, very small and unfortunately with all the job descriptions at my employer I wasn’t eligible for employment anymore. … I busted my butt at that place and gave it everything that I had, even very sick and ill and not well and they, I feel like they tossed me in the trash.”*

When another participant started a new job as a nurse, she found her new employer was unwilling to accommodate job duty recommendations from her healthcare provider:

*“I told her like maybe on day seven that I had a nurse’s note and when she told me that she was not going to accommodate it, I just basically let her know like, ‘well I can’t continue to work here. Like I need to,’ you know in the note it says like okay well, ‘I need to be able to take frequent breaks like to sit down, I can’t stand for longer than two hours, I should be able to take water breaks, bathroom breaks and stuff like that.’… She had a whole attitude about it and told me that she was not going to accommodate my nurse’s note so I was like ‘okay, you’re not going to accommodate my nurse’s note like so you want me to put myself and my baby in danger; no thank you, you can keep this job*.’”

Ultimately, the participant quit the new job because the employer would not accommodate the requests in her healthcare provider’s note.

## Discussion

We present novel qualitative data that provides insights into US women’s experiences balancing a pregnancy at increased risk for preterm birth (PTB) with employment obligations. The participants were majority non-Hispanic Black (NHB), as NHBs bear a disproportionate burden of PTB within in our clinic and across the nation. Additionally, the majority of women in the study were publically insured and employed in occupations that are most often low income. While many women reported positive experiences, the most striking insights came from women who described negative situations that ranged from challenging to potentially unlawful.

Employment status changes including voluntary and involuntary reduction of hours and termination were some of the most notable findings of the study. Multiple participants described voluntarily reducing their work hours or terminating their employment due to physical discomfort or perceived risks their employment posed to pregnancy. These findings may represent women taking bold measures to advocate for their health during pregnancy. However, in most cases, even during high risk pregnancy, it is considered safe and healthy for women to continue working during pregnancy. A meta-analysis evaluating the impact of work activities on pregnancy outcomes including prematurity, low birth weight and hypertensive complications found increasing evidence to suggest that employment-related risks to pregnancy are minimal [8]. Based on these and other data, the American College of Obstetricians and Gynecologists (ACOG) recommends that it is “generally safe” for women to work during pregnancy [3]. Notably, most of these data do not directly address women with a history of PTB. Although the data is limited in this setting, most experts agree that a history of PTB alone does not necessitate activity restriction such that women are unable to work.

Simple modifications such as a sitting stool or more frequent breaks often allow a woman to safely and more comfortably complete her work duties during a high risk pregnancy [9].

Accommodations to limit standing and lifting may be indicated in physically demanding professions, as there are data to suggest lifting cumulative weight over 100 kg per day and standing for over three hours per day increases the risk of PTB [10, 11]. Many of the participants in our study reported positive experiences with employers who proactively made simple accommodations to facilitate continued safe and comfortable employment during pregnancy. Other participants described employers who were unable and/or unwilling to modify job duties, leading to loss of employment. The Americans with Disabilities Act requires employers to provide “reasonable” accommodations for pregnancy-related impairments, as long as it does not cause “undue hardship” to the employer [3]. This legislation requires employers to provide accommodations for pregnant women that would be rendered to other employees with a time-limited medical impairment. For example, if an employee was accommodated with a sitting stool after a back injury, a pregnant women with a similar job description should also have access to a sitting stool. However, the Americans with Disabilities Act only applies to employers with 15 or more employees and is similarly challenging to enforce [3].

Perhaps the most concerning disclosures were women who reported involuntary reduction in their employment hours and coercion to prompt resignation. Although it is important to note that the data presented represents events solely from the patient perspective, without insights from the employer or provider, an involuntary reduction in employment hours simply due to pregnancy is unlawful in many circumstances. The US Pregnancy Discrimination Act prohibits forcing a pregnant woman to take leave, who is otherwise capable of performing her work functions [3]. However, the protections offered by this legislation are limited. This legislation only applies to specific employers (i.e. employers with over 15 employees) and can be difficult to enforce, as one must file a claim with the Equal Employment Opportunity Commission when there is an infringement [3].

Our study presents important insights from the patient perspective about their experiences balancing employment with high risk pregnancy. Despite the impact of our findings, important limitations must be considered. The original intent of the IDIs was not focused solely on employment. Given that the parent study was broader in scope, the IDI guide omitted key follow-up probes to employment-related questions that could have yielded even greater insights on this topic. For example, many participants described situations where it is unclear if the physical limitations in the workplace were based on recommendations from a medical provider or based on the participant’s own comfort level. Additionally, due to logistical limitations, our study excluded non-English speaking women. Non-English speaking women in the US may be the most vulnerable to challenging work conditions with limited ability to modify their job duties in the setting of high risk pregnancy. Women who identify as Hispanic ethnicity were outside of the scope of the original research aims of the parent study; therefore, the undoubtedly valuable insights from this patient population are not represented in the current study. Further studies incorporating Hispanic and non-English speaking women are urgently needed. The data in the current study also include participants recruited from a single academic institution and therefore may have limited generalizability.

The majority of participants in our study are non-Hispanic Black and publically insured. Although it is unknown whether their experiences reflect the broader experiences of pregnant women across the US, NHB women in our clinic and across the US bear a disproportionate burden of PTB [5] and more commonly work in lower-income, less flexible occupations [4]; therefore their insights into employment barriers within the setting of high risk pregnancy care are essential. The literature has documented that adherence to PTB prevention strategies is often uniquely challenging for NHB women [12, 13]. For example, despite recommendations from the American College of Obstetricians and Gynecologists (ACOG) supporting weekly injections of 17-hydroxyprogesterone caproate (17-P) to reduce the risk of recurrent PTB, multiple studies document reduced adherence to 17-P in NHB women [12, 13]. Although the efficacy of 17-P is being re-evaluated, it was once the cornerstone of PTB prevention; therefore, much of the published work on adherence to PTB prevention focuses on 17-P. In a study of women who previously delivered preterm, patients identified limited time-off from work and inflexible work schedules as barriers impeding adherence to the weekly 17-P injection schedule [14]. The findings within the 17-P literature surrounding adherence highlight the urgent need for uncovering and addressing barriers to adherence that are culturally sensitive and inclusive of women from racial and ethnic backgrounds most impacted by PTB.

The impact of PTB is also disproportionate among women with lower socio-economic status [5] and most of the women in our study work in occupations that are traditionally lower-paying. While the interview guide did not inquire directly about income or educational attainment, given the high number of women in the study on public insurance, we suspect that many likely had lower incomes and less formal education. Participants were also employed in two of the most common occupations held by women in general (healthcare/nurse and customer service), four of the most common occupations held by pregnant women (food service, retail, healthcare/nurse and customer service), and the three of the most commonly held occupations by pregnant Black women (retail, healthcare/nurse and customer service) [4, 7]. Although the experiences recounted may not be representative of all pregnant women at high risk for PTB, the findings suggest the workplace environment could be improved, thus mitigating barriers for pregnant women working across an array of commonly held occupations.

Our findings highlight important opportunities to mitigate barriers to high risk prenatal care and potentially improve adherence to PTB prevention strategies through education. Many of the findings suggest that profound misunderstandings likely exist at the patient, employer and provider level about the laws surrounding employment in pregnancy, the medical risks of continued employment, and the range of creative accommodations that often allow for continued workplace productivity even during high risk pregnancy. To address this need, we have established a collaboration with the Duke Health Justice Clinic to develop trainings and educational materials. Future studies using educational interventions aimed at patients, employers and medical providers to improve understanding about appropriate limitations and accommodations may be uniquely impactful to improve timely initiation of and adherence to high risk prenatal care and PTB preventive care plans.

## Conclusions

Women balancing pregnancy at high risk for preterm delivery with employment obligations report a variety of experiences. While some women reported positive experiences, many women described situations that ranged from challenging to potentially unlawful. Our findings suggest that profound misunderstandings likely exist at the individual, employer and provider level about the laws surrounding employment in pregnancy, safe employment responsibilities during pregnancy, and the range of creative accommodations that often allow for continued workplace productivity even during high risk pregnancy. Future interventions aimed at individuals, employers and medical providers to improve understanding about appropriate modifications and accommodations may be uniquely impactful to improve timely initiation of and adherence to high risk prenatal care and preterm birth preventive care plans.

## Supplementary information

**Additional file 1.** Interview Guide Questions Focused on Employment.

## Data Availability

Data sharing is not applicable to this article as no datasets were generated or analyzed during the current study.

## Abbreviations

IDIs: In-depth interviews
NHB: Non-Hispanic Black
PTB: Preterm birth

## References

[1] Murphy SL, Xu J, Kochanek KD, Arias E. Mortality in the United States, 2017. NCHS Data Brief. 2018;328(328):1–8. 30500322

[2] Osterman MJK, Martin JA (2018). Timing and adequacy of prenatal Care in the United States, 2016. Natl Vital Stat Rep.

[3] Committee Opinion No ACOG (2018). 733: employment considerations during pregnancy and the postpartum period. Obstet Gynecol.

[4] Harwood MH (2019). Sarah David by the numbers: where do pregnant women work?.

[5] 2019 March of Dimes Report Card. 2019; https://www.marchofdimes.org/mission/reportcard.aspx#:~:text=2019%20MARCH%20OF%20DIMES%20REPORT,to%2010.02%20percent20in202018.&text=Choose%20your%20state%20to%20see,on%20this%20year's%20Report%20Card.

[6] Guest G, MacQueen KM, Namey EE (2012). Applied thematic analysis.

[7] U.S. Department of Labor WsB. Most Common Occupations for Women in the Labor Force 2017; https://www.dol.gov/agencies/wb/data/employment-earnings-occupations.

[8] Palmer KT, Bonzini M, Harris EC, Linaker C, Bonde JP (2013). Work activities and risk of prematurity, low birth weight and pre-eclampsia: an updated review with meta-analysis. Occup Environ Med.

[9] Satterfield N, Newton ER, May LE (2016). Activity in pregnancy for patients with a history of preterm birth. Clin Med Insights Womens Health.

[10] Magann EF, Evans SF, Chauhan SP (2005). The effects of standing, lifting and noise exposure on preterm birth, growth restriction, and perinatal death in healthy low-risk working military women. J Matern Fetal Neonatal Med.

[11] Cai C, Vandermeer B, Khurana R (2020). The impact of occupational activities during pregnancy on pregnancy outcomes: a systematic review and metaanalysis. Am J Obstet Gynecol.

[12] Yee LM, Liu LY, Sakowicz A, Bolden JR, Miller ES (2016). Racial and ethnic disparities in use of 17-alpha hydroxyprogesterone caproate for prevention of preterm birth. Am J Obstet Gynecol.

[13] Timofeev J, Singh J, Istwan N, Rhea D, Driggers RW (2014). Spontaneous preterm birth in African-American and Caucasian women receiving 17alpha-hydroxyprogesterone caproate. Am J Perinatol.

[14] Wheeler SM, Massengale KEC, Blanchard KP (2019). Improving uptake and adherence to 17-Hydroxyprogesterone Caproate in non-Hispanic black women: a mixed methods study of potential interventions from the patient perspective. Biores Open Access.

