# Supplementary material for: Pregnancy vs. paycheck: a qualitative study of patient’s experience with employment during pregnancy at high risk for preterm birth
Source: BMC Pregnancy Childbirth. 2020 Sep 25;20:565. doi: 10.1186/s12884-020-03246-7 (PMC7517633; doi:10.1186/s12884-020-03246-7)
Supplement: Supplementary file 1 — Additional file 1. Interview Guide Questions Focused on Employment. [file 12884_2020_3246_MOESM1_ESM.docx]

**Supplementary File. Interview Guide Questions Focused on Employment**

**In Depth Interview Guide**

| 1. Interviewer Name |  |
| --- | --- |
| 1. Participant ID# |  |
| 1. Pregnancy History (# prior pregnancies, #preterm, any pregnancy losses) |  |
| 1. Interview Context:    1. 🞏 Early presenter 🞏 Late presenter    2. 🞏 Non-Hispanic Black 🞏 Non-Hispanic White    3. 🞏 Scheduled interview 🞏 on-demand interview    4. Coordinated with clinic appointment? 🞏 Yes 🞏 No   If yes:  Appointment time (hhmm-24hr clock) \|___\|___\|___\|___\|  Appointment activities 🞏 Provider visit 🞏 Ultrasound 🞏 Nurse visit  Participant clinic arrival time (hhmm-24hr clock) \|___\|___\|___\|___\|  e. Other pre-interview comments: | |
| 1. Interview Date (mm/dd/yyyy) | \|___\|___\|/\|___\|___\|/\|___\|___\|___\|___\| |
| 1. Participant agrees for interview to be digitally recorded | Yes 🞏  No 🞏 |
| 1. Time Interview Began (hhmm-24hr clock) | \|___\|___\|___\|___\| |
| 1. Time Interview Ended (hhmm-24hr clock) | \|___\|___\|___\|___\| |

**Step 1:** Complete Q1-4 above before the interview.

**Step 2:** At the beginning of the interview, introduce yourself; thank participant for taking part in the interview.

**Step 3:** Read Section I below to participant.

**Step 4:** Ask participant’s permission to record interview; tick appropriate box in Q4 above.

**Step 5:** Turn on audio recorder if acceptable, document time interview begins in Q6 above, and
conduct interview.

**Step 6:** At the end of the interview, thank the participant and ask if she has any further questions; document time interview ended in Q7 above.

**Step 7:** Ask if the participant is interested in being re-contacted with study results; if yes, document appropriate email. Inform participant that her email address will not be linked with her study data.

***Interviewer: Please read the following to participants at the beginning of the interview.***

- Hello, thank you for taking time out of your busy schedule to speak with me today. My name is [Name], and I am an interviewer with the Preterm Birth Prevention study. [IF A PHONE INTERVIEW ASK: Is now still a good time to talk?]
- Before we begin, I’d like to tell you more about this interview and the research we’re doing.
- We are interviewing women with a history of preterm birth. Preterm birth happens when a woman gives birth before she is 37 weeks pregnant. A typical pregnancy lasts 40 weeks.
- Participating in this interview is voluntary. You can choose not to answer a question, or you can stop the interview at any time.
- With your permission, I would like to audio-record the interview. This is so I can remember everything that you say. The audio-recording will be stored on a secure server and destroyed after the findings of this research are published.
- If you do not want the interview audio recorded, I will take detailed notes throughout the interview instead. Your name will not be written on the notes or in the transcript produced from the recording. When we publish the findings, information from all participants will be grouped together, and your name will not be listed.
- The interview will take up to 1.5 hours. You will receive a $20.00 gift card and a gift bag for taking part.
- I am not a medical doctor or a medical provider. My role is to ask you about her feelings and experiences. If you have medical questions or concerns, I will help you find someone who is trained to manage those issues.
- Do you have any questions for me at this point?

Is it okay if I turn on the audio recorder now?

***[If yes, begin audio recording now.]***

***[If no]*** That’s okay, I’ll take detailed notes as we talk.

OK, let’s get started!

INTERVIEWER: Thanks again for your time! I would like to start off with general questions to get to know you a bit better.

Do you work outside the home?

*[If no]* Were you working when you found out you were pregnant with your current pregnancy?

What is your daily schedule like/what keeps you busy during the day?

*[If yes]* What kind of work do you do?

[Probe about structure of workplace if not volunteered e.g: Do you have a direct supervisor/ what does your day look like?

*[If works outside the home]*

*I want to talk a little bit about your job and specifically your experiences talking to your supervisor/boss at work.*

*First, do you have a supervisor or a boss? Is he/she in charge or your schedule?*

Have you told your supervisor about your pregnancy?

*[If yes]*

- When did you tell your supervisor about your pregnancy? (i.e. how long after you found out you were pregnant?)
- What was your supervisor’s reaction to your pregnancy? *[Probe about support or lack of support, ask for examples of support/no support]*

*[If no]*

- Please tell me more about your decision to not tell your supervisor about your pregnancy right now. *[Probe about when might tell employer]*

What kind of programs or support, if anything, does the company/boss/supervisor [pick the best term based on the participant’s description of her job] offer to women?

1. During pregnancy?
2. After pregnancy?

Did you have to take any time off work for your first visit?

*[If yes]* How did taking time off work affect your life? What about the rest of your prenatal care?

*[Probes, as appropriate]:*

- How much time did you have to take off?
- How did your employer react?
- Did you have to use vacation time or unpaid time off?
- Did your employer let you change your work hours so you could go to the clinic visit without having to take any time off?

Is there anything else you’d like to share with me before we end?

*[REMINDER: Ask the participant if she is interested in being re-contacted with the study results, and if so, collect e-mail address/inform that her email address will not be linked with study data.]*
